# King's College Hospital

**Published:** 1893-07-29

**Authors:** 


### FESTIVAL DINNERS AND MEETINGS.

### KING'S COLLEGE HOSPITAL.—SPECIAL MEETING.

The meeting held at Grosvenor House on Thursday, 20th inst., in aid of the funds of King's College Hospital, was largely attended, and nearly £2,700 was the immediate result of the special appeal for a five years' maintenance fund.

The present impoverished condition of this admirable institution is deeply to be regretted, and the possibility that wards may have to be closed is one that nobody can wish to see realized. Such a poor district as that in which King's College Hospital is situated naturally keeps the beds incessantly occupied, and when wards were closed on a former occasion the consequences were very disastrous. Poor breadwinners, always apt to put off coming to the hospital until the last possible moment, when they arrived seriously ill, had to be told that there were no beds available. What such words mean in the ears of these poor suffering men or women it is not difficult to imagine. King's College Hospital is making a brave struggle against any repetition of this past necessity, and numerous liberal responses will surely continue to be received to the appeal. The Duke of Westminster's own donation of £500 was an excellent example to set before those present at the meeting, and his Grace also bore witness to the perfectly efficient state of the hospital, and said that it would never do to allow wards to be closed in such an institution.

H.R.H. the Duke of Cambridge, President of the Hospital, spoke at some length, and dwelt on the fact that even when all the beds were available there was great difficulty in meeting all the cases requiring admission. It was essential for those who were connected with the management to make every effort to keep the institution going in the condition in which it was at the present time. Unless, however, great efforts were made in that direction, it would be impossible to do it. As they had already heard, the funds of the institution were eaten up, they had unfortunately been obliged to avail themselves of all the legacies that had been left them, in addition to which they were in debt to the extent of £6,000, which was undeniably a very serious state of affairs. The real fact was that the hospital had only £1,000 a year which it could rely upon, everything else that was required must come from the public, either in the way of subscriptions or donations. The annual subscriptions amounted to an average of only £2,000, the donations to £4,500, and the grants from the Saturday and Sunday Funds to about £1,700. Its income, therefore, from ordinary sources, amounted to little more than £9,000. In order to fulfil the indispensable requirements of a Metropolitan General Hospital it was necessary for them to expend not less than £16,500 a year. He had every reason to believe that the public generally had the greatest confidence in the hospital that it was well conducted, and its funds well and judiciously administered. It was quite true that large sums had been expended in the development of their medical schools, but by that means they were enabled to produce good and efficient medical students, who, when they went out into the world, were able to perform their duties, not only in a manner creditable to themselves, but of immense advantage to the public interests in general. These results could only be obtained by maintaining their medical schools in the greatest state of efficiency.

The Bishop of London urged the public to increase their subscriptions more than donations, by which the hospital might be able to depend upon some permanent support. Latterly the hospital had been maintained by legacies, which were a fluctuating source of income, and if the charitably disposed would guarantee them against fluctuations for, say, the next ten years, it would be of great advantage to the institution.

The Hon. W. F. D. Smith, M.P., urged the need for subscriptions being raised to enable the hospital to maintain its present highly satisfactory condition.

Mr. Richard Twining, the Treasurer, made special mention of the perfection of the nursing arrangements, and asked for sufficient help "to give us tranquil minds for the next five years."

Sir William Priestley was followed by Mr. Horton Smith, M.P., who said that the hospital committee, of which he was a member, was constantly engaged in the experiment of making £9,000 a-year cover the expenses, which amounted to about £18,000, and although it was not an easy thing to do he thought they succeeded pretty well.

The Rev. Dr. Wace, Chairman of the Committee of Management, spoke warmly of the constant interest which the Duke of Westminster evinced in the hospital, and of the benefit he had conferred by the loan of Grosvenor House on the present occasion. It is to be hoped that so well-attended and enthusiastic a meeting will have a permanently beneficial influence.

